# Human Th17 cells can be induced through head and neck cancer and have a functional impact on HNSCC development

**DOI:** 10.1038/sj.bjc.6605891

**Published:** 2010-09-28

**Authors:** R Kesselring, A Thiel, R Pries, T Trenkle, B Wollenberg

**Affiliations:** 1 Department for Otorhinolaryngology, University of Luebeck, Ratzeburger Allee 160, Luebeck 23538, Germany; 2 Department for Oral and Maxillofacial Surgery, University of Luebeck, Ratzeburger Allee 160, Luebeck 23538, Germany

**Keywords:** Th17, HNSCC, tumour-infiltrating lymphocytes, IL-17, tumour microenvironment

## Abstract

**Background:**

The T helper 17 (Th17) cells recently identified as distinct T helper cell lineage are characterised by their production of the proinflammatory cytokine interleukin 17. Although much effort has been made in understanding the function of Th17 cells in the pathogenesis of different diseases, their influence in carcinogenesis remain largely unknown.

**Methods:**

We studied the prevalence and induction of Th17 cells in head and neck squamous cell carcinoma (HNSCC) patients by flow cytometry. To determine the migration mechanism of Th17 cells into primary tumours and metastasis of HNSCC, we performed chemotaxis assays. We analysed the proliferation and the angiogenesis-related proteins of HNSCCs in the presence of Th17 cells with MTT-based proliferation assay and an angiogenesis protein array.

**Results:**

In this study, we showed that the prevalence of Th17 cells is elevated in peripheral blood of HNSCC patients. In addition, tumour tissue and tumour-draining lymph nodes are infiltrated by a huge number of Th17 cells representing an important fraction of the tumour-infiltrating lymphocytes (TILs). We further showed that Th17 cells can be induced and expanded in tumour microenvironment through cytokines produced by tumour cells and TILs, and in addition can be recruited to the tumour milieu through a CCR6/CCL20-dependent mechanism. Furthermore, we showed that the proliferation and angiogenesis of HNSCC are impaired in the presence of Th17 cells.

**Conclusion:**

We conclude that Th17 cells have a substantial impact on the carcinogenesis of HNSCCs and on their metastasis and could serve as a potential therapeutic target to modulate anti-tumour response in HNSCC.

T helper 17 (Th17) cells are proinflammatory CD4+ effector T helper cells, which produce the cytokine interleukin 17 (IL-17). This distinct T helper cell lineage was discovered 5 years ago (Harrington *et al*, 2005) and have a prominent function in mucosal immunity and autoimmune diseases such as multiple sclerosis, as well as human inflammatory bowel disease and psoriasis. Aside from secreting IL-17A, human Th17 cells produce the cytokines IL-17F, -21, -22 and -26, and express the surface receptors CD161, CCR6, IL-23R (Cosmi *et al*, 2008; Singh *et al*, 2008) and the orphan nuclear receptor ROR2C (Ivanov *et al*, 2006). In addition to Th17 cells, there are subpopulations of CD8+ T cells, *γδ* cells and NKT cells able to produce IL-17 (Coquet *et al*, 2008; Lee *et al*, 2008; Rachitskaya *et al*, 2008; Roark *et al*, 2008; Kondo *et al*, 2009; O’Brien *et al*, 2009). The secretion of IL-17 leads to the induction of chemokines, matrix metalloproteinases and antimicrobial peptides in surrounding tissue cells, leading to inflammation and the recruitment of neutrophils and macrophages (Martinez *et al*, 2008). The differentiation of Th17 cells in human beings is induced through the cytokines IL-1*β* and -6 (Acosta-Rodriguez *et al*, 2007) and requires the transcription factor ROR2C (Ivanov *et al*, 2006); their expansion is regulated by IL-23 (Chizzolini *et al*, 2008). Several cytokines, including type I and type II interferon, IL-2, -4, -12 and -27 inhibit Th17 differentiation (Diveu *et al*, 2009). The Th17 cells are an ambitious field in current immunology research, but less is known about the function of Th17 cells in cancer development, particularly nothing is known about the Th17 lineage in head and neck squamous cell carcinomas (HNSCCs).

Head and neck cancer is an aggressive malignancy comprising about 6% of all new diagnosed cancers (Liu *et al*, 2009). A total of 95% of tumours arising in the head and neck region are squamous cell carcinomas. The HNSCCs are most commonly associated with smoking and alcohol abuse (Uppaluri *et al*, 2008). Despite advances in surgery, radio- and chemotherapy, HNSCC patients have a poor prognosis because of a high recurrence rate (Braakhuis *et al*, 2005). Although it was shown that HNSCCs are infiltrated by a high number of various species of tumour-infiltrating lymphocytes (TILs) (Uppaluri *et al*, 2008), these are not capable to eradicate the tumour as a result of evolving multiple mechanisms for tumour immune evasion by HNSCC tumours and deranging of the functions of TILs in HNSCC patients. It is assumed that HNSCCs produce various immunosuppressive and tumour-promoting cytokines, which leads to the impaired anti-tumour response (Pries and Wollenberg, 2006); however, the exact mechanisms, which leads to these immunomodulations, remain mostly unknown (Chin *et al*, 2004) and are discussed highly controversial. In addition, it is hypothesised that tumour cells develop mechanisms to evade growth inhibitory effects of cytokines that are present in the tumour microenvironment. It is well established that inflammation is closely connected to HNSCC development (Douglas *et al*, 2004), because of the induction of chronic inflammation caused by exposure to irritants in inhaled air, especially cigarette smoke, which enhance the nidation of viruses and airborne microbes and therewith promote tumour growth. In consideration of these facts, we propose a high impact of the proinflammatory Th17 cells on HNSCC pathogenesis and anti-tumour response.

In our study, we revealed that HNSCC patients have elevated levels of Th17 cells in their peripheral blood compared with healthy controls. In addition, huge numbers of Th17 cells infiltrate primary tumours as well as lymph node metastasis of head and neck cancer. We could identify the HNSCC microenvironment as a strong Th17 cell inducer. Further, we could show for the first time that Th17 cells actively migrate to the HNSCC tumour milieu and have a functional impact on tumour proliferation and angiogenesis in human beings.

## Materials and methods

### Samples

Peripheral blood was obtained from HNSCC patients (*n*=25, age=52–72 years, mean 62 years) and as controls from healthy individuals (*n*=25). All donors participated on a voluntary basis and gave written informed consent. This study was conducted according to the principles expressed in the Declaration of Helsinki and was approved by the local ethics committee. Tumour tissue (*n*=10) and metastatic lymph nodes (*n*=20) were obtained from head and neck cancer patients undergoing head and neck surgery. For HNSCC samples, patients with a known history of HNSCC were enrolled and malignancy was assessed by a pathological examination of the biopsies. Patients included in the study had not yet received any chemo- or radiotherapy.

### Isolation of T cells and Th17 cells

The PBMCs were isolated from freshly derived peripheral blood by centrifugation on a Ficoll Hypaque density gradient (PAA Laboratories, Pasching, Austria). The CD4+ cells were enriched by negative selection using a CD4 T cell isolation kit II (Miltenyi Biotec, Bergisch Gladbach, Germany) according to the manufacturer's instructions. The purity of T cells was >90% determined by flow cytometry. The Th17 cells were purified using an IL-17 Secretion Assay-Cell Enrichment and Detection kit (Miltenyi Biotec) according to the manufacturer's instructions and a postsort with an FACS Aria (BD Biosciences, Heidelberg, Germany). We revealed a purity of 98% gated on PI-negative cells.

### Cell culture and tumour supernatants

The permanent HNSCC cell lines, Pittsburgh Cancer Institute-1 (PCI-1), PCI-13 (generously given to us by Theresa Whiteside, Pittsburgh Cancer Institute) and BHY, were cultured in DMEM (PAA Laboratories) supplemented with 10% heat-inactivated FCS, 1% non-essential amino acids and 1% sodium pyruvate (PAA Laboratories) under standard culture conditions (37°C, 5% CO_2_). Tumour supernatants of these cell lines were collected 48 h after medium exchange and were immediately cryopreserved and stored in liquid nitrogen. The PBMCs and T cells were cultured in RPMI1640 (PAA Laboratories) supplemented with 100 U ml^–1^ penicillin, 0.1 mg ml^–1^ Streptomycin, 0.3 mg ml^–1^ L-glutamine (Sigma-Aldrich, Steinheim, Germany) and 10% human AB serum (PAA Laboratories) in 96-well plates (Nunc, Langenselbold, Germany) at 5 × 10^4^ cells per well.

### Single cell suspensions

Freshly derived tumour tissue or tumour-draining lymph node (TDLN) tissue was minced into very small pieces and washed in PBS. The tissue was digested in DMEM supplemented with 300 U ml^–1^ collagenase and 100 U ml^–1^ hyaluronidase (Sigma-Aldrich) for 2 h at 37°C under permanent rotating. Afterwards, the suspension was washed in PBS and resuspended in DMEM supplemented with 5 mg ml^–1^ dispase (Sigma-Aldrich) for 60 min. The cell suspension was filtrated through a 100 *μ*m filter to obtain the single cell suspension. For cytokine analysis, 10 *μ*g ml^–1^ Brefeldin A (Sigma-Aldrich) was added during all steps of incubation.

### Th17 cell induction

For the induction of Th17 cells, isolated CD4+ T cells were stimulated with 5 *μ*g ml^–1^ CD3 (OKT3) and 1 *μ*g ml^–1^ CD28 (CD28.2) antibody (BD Biosciences) in RPMI1640 with 50 U ml^–1^ IL-2 (eBioscience, Cambridge, UK) in 48-well plates (Nunc). The T cells were either cultured with tumour supernatants from HNSCC cell lines or from single cell suspensions of tumour or TDLN tissue or with IL-1*β* (10 ng ml^–1^), IL-6 (20 ng ml^–1^), IL-23 (10 ng ml^–1^) or medium alone. On day 7, the T cells were harvested, stained for flow cytometry analysis to quantify the induction of Th17 cells. For blocking experiments, blocking antibodies for IL-1*β*, -6, -23 and prostaglandin E(2) (PGE(2)) (R&D, 5 *μ*l ml^–1^) were added to the Th17 cells during incubation.

### Antibodies

Monoclonal antibodies (mAbs) were purchased from Becton Dickinson except for anti-human IL-17A, IL-22, CCR6, IL-17R, IL-21R, IL-22R antibodies obtained from R&D (Minneapolis, MN, USA) and anti-human CD27, IL-17A, -23 and ROR*γτ* obtained from eBioscience. All mAbs were used according to the manufacturer's recommendations.

### Flow cytometry

Before intracellular staining, T cells were restimulated for 4 h with 50 ng ml^–1^ phorbol 12-myristate 13-acetate (PMA) and 100 ng ml^–1^ ionomycin (Sigma-Aldrich) in the presence of 10 *μ*g ml^–1^ Brefeldin A. The cells were analysed on an FACS Canto A flow cytometer (BD Biosciences) with FACS Diva software 6.0 and evaluated with FlowJo software (TreeStar, Ashland, OR, USA). The T cells were surface stained according to standard protocols. Briefly, T cells were washed three times in FACS buffer (PBS supplemented with 1% BSA and 0.1% sodiumazide) and stained with fluorescent-labelled mAbs for 20 min at 4°C. For intracellular staining, cells were fixed and permeabilised with the Flow Cytometry Fixation and Permeabilisation Buffer kit (R&D) according to manufacturer's protocol and subsequently intracellularly stained. Fluorescent-labelled isotype controls (BD Biosciences) or fluorescence-minus-one controls were used in all experiments to determine background fluorescence and exclude unspecific binding. Doublets of cells were excluded in the analysis, as well as dead cells, which were excluded by Annexin V/PI staining (BD Biosciences). In case of the usage of unstained primary antibodies (IL-23), a control with secondary antibody without primary antibody was performed to detect unspecific binding of the secondary antibody.

### ELISA and multiplex cytokine assay (Luminex)

Secreted IL-23 was measured with the human IL-23(p19/p40) ELISA Ready-SET-Go! kit from eBioscience according to the manufacturer's protocol. Multiplex immunoanalytic xMAP technology was used for the measurement of cytokines in tumour supernatants. Samples were cryopreserved and stored in liquid nitrogen. Directly before analysis, the aliquots were thawed slowly on ice. Commercially available cytokine kits (Bio-Rad Laboratories, Munich, Germany) were used. The measurement was performed with the Bioplex 200 system and Bioplex manager software (Bio-Rad Laboratories). The analysis and preparation were performed according to the manufacturer's instructions.

### Immunofluorescence

Tumour tissue was embedded in Shandon Cryochrome (Thermo Electron Corporation, Pittsburgh, PA, USA) and maintained in liquid nitrogen. The tissue was cut into 6 *μ*m sections. The slides were fixed in ice-cold acetone for 10 min, air dried and washed in TBS (Bio-Rad Laboratories). The sections were incubated in Ultra Block (LabVision, Fremont, CA, USA) for 30 min to diminish unspecific binding. The primary mAbs mouse anti-human CD4 [1 : 100] and goat anti-human IL-17 [1 : 50] were diluted in staining buffer (TBS supplemented with 1% Ultra Block) and the sections were incubated overnight at 4°C in a humidified chamber. After three washing steps with TBS, secondary antibodies (Alexa Fluor 350-conjugated rabbit anti-goat IgG [1 : 100] (Invitrogen, Karlsruhe, Germany) and Cy2-conjugated rabbit anti-mouse IgG [1 : 400] (Dianova, Hamburg, Germany)) diluted in staining buffer were added for 1 h. The slides were counterstained with PI (10 *μ*l ml^–1^) for 3 min at room temperature. Isotype controls were performed for each antibody. The sections were visualised using a Zeiss Axiovert 200 M fluorescence microscope equipped with an Apoptome and Zeiss Axio Vision Software (Zeiss, Göttingen, Germany).

### Proliferation assay

The proliferation of tumour cells was determined with an MTT (3-(4,5-dimethylthiazol-2-yl)-2,5-diphenyltetrazolium bromide) assay (Sigma-Aldrich) according to the manufacturer's instructions and was evaluated with an iMark Microplate Absorbance Reader (Bio-Rad Laboratories).

### Migration assay

The migration of Th17 cells was studied performing a chemotaxis assay using a ChemoTx 96-well chamber with 5 *μ*m pore size (Neuroprobe Inc., Gaithersburg, MD, USA) and quantitated by flow cytometry. The wells of the ChemoTx plate were filled either with serum-free media or freshly derived single cell suspensions of tumour or TDLN tissue suspended in serum-free media. Isolated Th17 cells were transferred onto the filter of the chemotaxis chamber. The chamber was incubated for 7 h at 37°C. After incubation, the non-migrated cells were flushed with PBS and the number of migrated Th17 cells in the wells was determined. To block CCR6, a CCR6 blocking antibody (R&D, 5 *μ*l ml^–1^) was added to the cell suspensions 30 min before incubation of the chemotaxis assay.

### Angiogenesis/vasculogenesis protein array

The HNSCC cell line PCI-13 was incubated with isolated Th17 cells or PBMCs depleted from Th17 cells for 48 h. Afterwards, the protein of the tumour cells was isolated. Briefly, cells were scraped from the tissue culture flask and resuspended in lysis buffer (1% NP-40, 20 mM Tris–HCl (pH 8.0), 137 mM NaCl, 10% glycerol, 2 mM EDTA, 10 *μ*g ml^–1^ Aprotinin, 10 *μ*g ml^–1^ Leupeptin). After 45 min of incubation on ice, the suspension was centrifuged, the supernatant decanted and the amount of protein was measured photometrically. Angiogenesis-related protein expression was examined with the human angiogenesis antibody array (R&D) according to the manufacturer's protocol. The data was evaluated with the Quantity One software (Bio-Rad Laboratories) by quantifying the mean spot pixel densities.

### Statistical analysis

Standard two-tailed Student's *t*-tests were used for statistical analysis with *P*-values ⩽ 0.05 considered significant. Significant data sets are labelled with an asterisk (^*^). We evaluated the statistics with SPSS Statistics software (SPSS GmbH Software, Munich, Germany).

## Results

### The level of Th17 cells is elevated in peripheral blood of HNSCC patients

**Figure 1:**
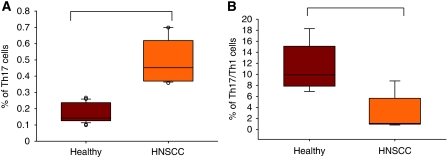
Prevalence of Th17 and Th1/Th17 cells in peripheral blood of HNSCC patients. The CD4+ T cells were isolated from peripheral blood of HNSCC patients and healthy controls and subsequently incubated for 4 h in the presence of PMA/Ionomycin and Brefeldin A. The cells were stained with CD3-FITC, CD4-PerCP, IL-17-APC, ROR2C-PE and IFN*γ*-PECy7 and analysed by flow cytometry. The CD3 and CD4+ T cells were gated in the FACS analysis. (**A**) Comparison of the prevalence of Th17 cells in peripheral blood of healthy persons (*n*=25) and HNSCC patients (*n*=25) with *P*<0.05. The data is expressed as the frequency of Th17 cells in the lymphocyte population. The box plots show the median (middle line), 25th and 75th percentiles (box), the extreme values (whiskers, which indicate the 90th and 10th percentile) and the outliers (circles). (**B**) Prevalence of Th17/Th1 cells in peripheral blood of head and neck cancer patients (*n*=25) compared with healthy controls (*n*=25) with *P*<0.05. The data is expressed as the frequency of Th17/Th1 cells in the Th17 population.

To address the issue whether there is a disturbed prevalence of Th17 cells in head and neck cancer patients, we first analysed the prevalence of Th17 cells in peripheral blood of HNSCC patients compared with healthy controls. The PBMCs were isolated and restimulated with PMA and ionomycin for 4 h in the presence of Brefeldin A and Th17 cells were quantified by flow cytometry. The Th17 cells were identified as CD3+CD4+IL-17+ cell population. As shown in Figure 1A, there was a statistically significant higher percentage of Th17 cells in peripheral blood of cancer patients (0.4568±0.0319%, *n*=25) compared with healthy controls (0.1574±0.0272%, *n*=25) with *P*<0.01.

### Th1/Th17 cells are decreased in peripheral blood of HNSCC patients

Th1/Th17 cells, which simultaneously produce the Th17 cytokine IL-17 and the Th1 cytokine IFN*γ*, account for approximately 10% of the whole Th17 cell population in healthy individuals (11.33±1.437%). In peripheral blood of HNSCC patients, this subpopulation is significantly decreased (1.14±1.113%) with *P*<0.05 (Figure 1B). We identified the Th1/Th17 cells as the CD3+CD4+IL-17+IFN*γ*+ population.

### Tumour tissue and TDLNs are infiltrated by a huge number of Th17 cells

**Figure 2:**
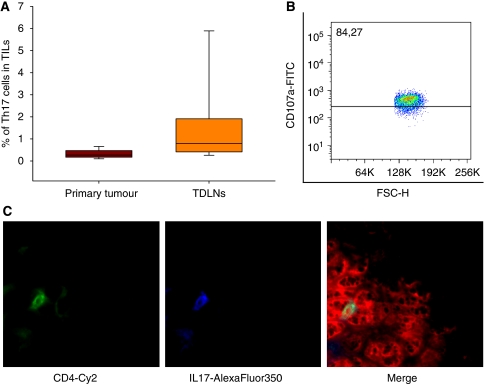
Identification of Th17 cells in primary tumours and TDLNs of HNSCC patients. Tumour and lymph node tissues were enzymatically digested to obtain single cells suspension. These cells were stained with CD3-FITC, CD4-PerCP, IL-17-APC and ROR2C-PE analysed by flow cytometry. (**A**) Frequency of the Th17 cells in primary tumours (*n*=10) and lymph node metastasis (*n*=15). The box plots show the median (middle line), 25th and 75th percentiles (box), the extreme values (whiskers, which indicate the 90th and 10th percentile) and the outliers (circles). (**B**) Flow cytometric analysis of tumour-derived Th17 cells for their CD107a expression. The cells were stained with CD107a- FITC, CD3-APC Cy7, CD4-PerCP, IL-17-APC. (**C**) Immunhistochemical staining of tumour and TDLN tissue. The Th17 cells were stained with mAbs against IL-17 (secondary conjugated with AlexaFluor 350) and against CD4 (secondary labelled with Cy2) and counterstained with PI as described in the Materials and methods. Control staining without primary antibodies and isotype controls are negative. The three figures show first the single fluorescence channels (first column=AlexaFluor 350; second column=Cy2) and the last column shows the overlay of all three channels (blue, red, green).

Next, we determined the amount of Th17 cells infiltrating tumours and metastatic lymph nodes of head and neck cancer. In 100% of the TDLNs, we could identify a high proportion of CD4+IL17+ cells (0.2345±0.0584% of all TILs) (Figure 2A and B). We could also detect Th17 cells in primary HNSCCs of all locations (0.8467±3.567% of all TILs and 10.65%±3.567 of all T cells) except for larynx carcinomas, which were sparely infiltrated by lymphocytes and so we were not able to detect a definite Th17 cell population. To affirm that these Th17 cells actually secrete IL-17 in tumour milieu, we measured the IL-17 concentration from tumour supernatants derived from tumour single cell suspensions by ELISA. Indeed, we measured an IL-17 concentration of 512±175 pg ml^–1^ (*n*=10) (data not shown).

### Th17 cell inducing cytokines IL-1*β*, -6 and -23 are present in HNSCC tumour milieu

**Figure 3:**
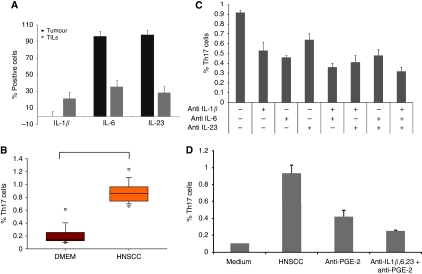
Induction of Th17 cells in HNSCC microenvironment. (**A**) Expression of the Th-17-promoting cytokines IL-1*β*, -6 and -23 in tumour cells and TILs *ex vivo* analysed by flow cytometry. The bars show the mean of the positive cells±s.d. (**B**) Induction of Th17 cells in HNSCC microenvironment. T cells were incubated with tumoursupernatants from single cell suspension of tumour tissue for 7 days (*n*=30) with *P*<0.05. The cells were stained with CD3-FITC, CD4-PerCP, IL-17-APC and analysed by flow cytometry. The data is expressed as the frequency of Th17 cells in the CD4+ T cell population. The box plots show the median (middle line), 25th and 75th percentiles (box), the extreme values (whiskers, which indicate the 90th and 10th percentile) and the outliers (circles). (**C**) Blocking of Th17-inducing cytokines with blocking antibodies diminishes the Th17 induction through HNSCC tumourmilieu. T cells were incubated with tumoursupernatants from single cell suspension of tumour tissue for 7 days in the presence of blocking antibodies (*n*=15). First column cytokines are not blocked, columns 2–4 block one of the cytokines IL-1*β*, -6, -23, columns 5–7 block two of the Th17 cell-inducing cytokines, column 8 block all three cytokines. The error bars show the s.d. (**D**) PGE(2) contributes to Th17 cell induction in HNSCC microenvironment. T cells were incubated with tumoursupernatants from single cell suspension of tumour tissue for 7 days in the presence of a PGE(2) blocking antibody (*n*=15). Blocking of PGE(2) (column 3) leads to a decrease in Th17 induction in HNSCC milieu. Blocking of IL-1*β*, -6, -23 and PGE(2) leads to a significant downregulation of Th17 cells compared with the number of Th17 cells induced through tumour supernatants alone. The error bars show the s.d.

We investigated whether the increased prevalence of Th17 cells in tumours and TDLNs is caused by an induction of Th17 cells or by the migration of Th17 cells towards the tumour site. The differentiation of Th17 cells in human beings is initiated and regulated by the cytokines IL-1*β* and -6 and their maintenance by IL-23 (Romagnani *et al*, 2009). Thus, to examine the first hypothesis, we determined the existence of these cytokines in HNSCC tumour milieu. The cytokines in tumour supernatants were determined with ELISA and multiplex assays. We found that HNSCC cell lines secrete the cytokines IL-6 and -23, but not IL-1*β*. In tumour supernatants of freshly derived tumours and TDLNs, we could detect all three cytokines IL-1*β*, -6 and -23 (data not shown). To evaluate which type of cells from the single cell suspensions secrete the corresponding cytokine, we determined the cytokines also directly in *ex vivo* derived tumour cells and tumour-infiltrating cells by flow cytometry. The tumour cells secrete the cytokines IL-23 (99%) and IL-6 (99%). The IL-1*β* is secreted only by TILs (26%), which produce IL-23 (29%) and IL-6 (32%) as well (Figure 3A).

### Th17 cells can be induced through HNSCC tumour milieu

Subsequently, we analysed whether Th17 cells can be induced in HNSCC microenvironment. Supernatants from single cell suspensions of primary tumours as well as lymph node metastasis were collected 48 h after medium exchange and isolated T cells of healthy donors were incubated for 7 days with these supernatants (50% HNSCC supernatant+50% DMEM supplemented with IL-2) under TCR engagement. Thereafter, the frequency of Th17 cells was measured by flow cytometry. The T cells stimulated in DMEM show a statistically significant lower level of Th17 cells (0.1875%±0.0584) than T cells cultured with tumour supernatants from the primary tumours and TDLNs (0.8841%±0.136) (Figure 3B). To verify that the cytokines IL-1*β*, -6 and -23 are really the cause for the Th17 cell induction in HNSCC, we performed the above experiment with blocking antibodies for IL-1*β*, -6 and -23. We observed a reduction of the induction of Th17 cells when incubated with any of these blocking antibodies and especially for combinations of the antibodies (Figure 3C). Blocking of IL-1*β* yields a reduction in Th17 cell induction of 0.39%, blocking of IL-6 one of 0.47% and blocking of IL-23 one of 0.26%. A combination of blocking of the cytokines IL-1*β* and -6 leads to a reduction of Th17 cell induction of 0.54%, blocking of IL-1*β* and -23 one of 0.51% and blocking of IL-6 and -23 one of 0.42%. Blocking of the cytokines IL-1*β*, -6 and -23 yields a reduction of Th17 cell induction of 0.60%. However, the blocking of all three Th17-inducing cytokines could not abolish the induction of Th17 cells through HNSCC completely. Therefore, we investigated whether there are further factors in the tumour milieu, which lead to the Th17 cell induction. There are reports that Prostaglandin E2 is also involved in Th17 differentiation; therefore, we studied the effect of blocking of PGE(2) on Th17 cells induction in the presence of HNSCC supernatants. With a blocking antibody for PGE(2), there is a reduced Th17 cell induction (0.52%±0.082) compared with non-blocked induction (0.89%±0.136) (Figure 3D). Blocking of IL-1*β*, -6, -23 and PGE(2) yields the most prominent decrease of Th17 induction in HNSCC milieu (0.29%±0.03) (Figure 3D).

### Th17 cells migrate towards HNSCC milieu

**Figure 4:**
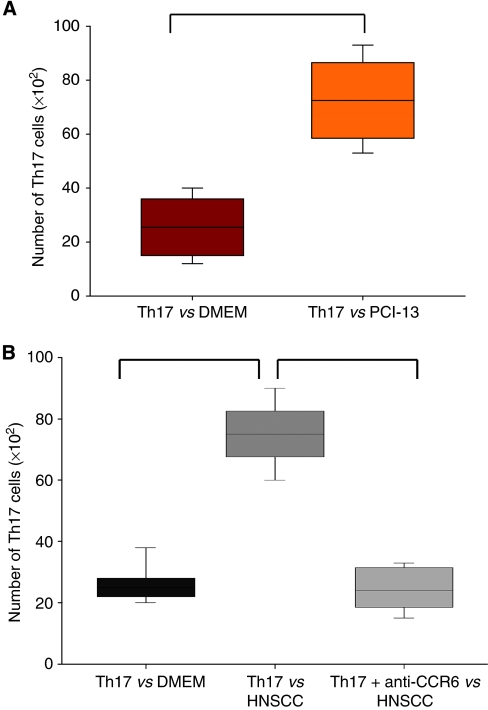
The Th17 cells migrate towards HNSCC milieu. (**A**) Migration assay with the ChemoTx Sytem as described in Materials and methods. In the wells of the 96-well plate, 20 × 10^3^ HNSCC cells of the cell line PCI-13 were seeded and on the top of the filter 20 × 10^3^ Th17 cells. After 8 h of incubation, the cells in the well of the 96-well plate were harvested and stained with CD3-FITC, CD4-PerCP, IL-17-APC and analysed with flow cytometry. The first column shows the migration of Th17 cells *vs* DMEM, the second column shows the migration of Th17 cells *vs* HNSCC cells (*n*=25) with *P*<0.05. The box plots show the median (middle line), 25th and 75th percentiles (box), the extreme values (whiskers, which indicate the 90th and 10th percentile) and the outliers (circles). (**B**) The migration of Th17 cells towards tumour is CCR6/CCL20 dependent. The Th17 cells were incubated with CCR6 blocking antibody. In the chemotaxis assay, these blocked Th17 cells (column 3) show a reduced migration towards HNSCC compared with the unblocked Th17 cells (column 2) (*n*=15) with *P*<0.05.

To test the second hypothesis that Th17 cells maybe able to migrate directly to tumour site, we performed a migration assay with a ChemoTx 96 chamber. The wells were filled with 20 × 10^3^ cells of the single cell suspension from tumour or TDLN tissue suspended in serum-free medium. A total of 20 × 10^3^ Th17 cells were applied on the top of the filter. After 7 h of incubation, we counted the migrated Th17 cells. We could detect an enhancement of migration towards the HNSCC filled wells (78 × 10^2^±861 migrated Th17 cells) compared with the DMEM filled wells (22 × 10^2^±569 migrated Th17 cells) with *P*<0.01 (Figure 4A). Owing to the fact that CCR6 is a surface receptor of Th17 cells, we next analysed whether the migration is CCR6/CCL20 driven. Hence, we incubated the tumour cells with a CCR6 blocking antibody 30 min before the incubation of the migration assay. We showed a diminished migration of CCR6-blocked Th17 cells towards the HNSCC cells (21 × 10^2^ migrated Th17 cells) compared with unblocked Th17 cells (78 × 10^2^±861 migrated Th17 cells) (Figure 4B).

### Th17 inhibits proliferation of HNSCC cells

**Figure 5:**
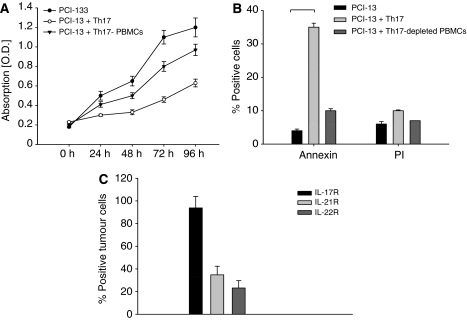
The proliferation of HNSCC cells is reduced in the presence of Th17 cells. (**A**) PCI-13 cells were incubated with Th17 cells, Th1 cells and DMEM in a transwell chamber over 96 h and the cell number was checked every 24 h with MTT reagents. The lines are the mean of eight-fold repeats±s.d. (**B**) The HNSCC cell line PCI-13 were incubated with Th17 cells, Th17-depleted PBMCs and DMEM, and after 24 h of incubation, the PCI-13 cells were harvested and stained with Annexin and PI and analysed by flow cytometry. The HNSCC cells incubated with Th17 cells show a significant upregulation of Annexin V as well as PI (*n*=15). The bars show the mean of the positive cells±s.d. with *P*<0.05. (**C**) Expression of IL-17R (first bar), IL-21R (second bar) and IL-22R (third bar) on tumour cells analysed by flow cytometry. The bars show the mean of eight-fold repeats±s.d. (**D**) PCI-13 cells were incubated with Th17 cells, Th17 cell supernatant and DMEM over 96 h and the tumour cell number was checked every 24 h with MTT reagents. The lines are the mean of eight-fold repeats±s.d.

Next, we considered whether the presence of Th17 cells in tumour has an impact on tumour growth. We performed an MTT assay to study the proliferation of tumour cells under the influence of Th17 cells. After 24 h incubation of the PCI-13 cells with the Th17 cells in a transwell chamber, there is an evident attenuation of the proliferation of HNSCC cells. We have tested different concentration of Th17 cells (Th17 cells : HNSCC cells 1 : 1, 5 : 1, 10 : 1) (Figure 5A). The data of the 10 : 1 and 1 : 1 concentrations are not shown. We have repeated this test three times with an eight-fold replication per test. With all three concentrations, there is a reduction in tumour growth, which is enhanced with higher concentration.

Concomitantly, we have measured apoptosis and necrosis of the tumour cells; when incubated together with Th17 cells, we could detect a statistically significant increase of Annexin V and PI staining after 24 h in tumour cells incubated with Th17 cells (33.0% Annexin V positive and 10.8% PI positive) *vs* tumour cells alone (3.9% Annexin V positive and 6.7% PI positive), whereas the apoptosis and necrosis in case of the PBMC population without Th17 cells are significantly lower (10.8% Annexin V positive and 6.8% PI positive) with *P*<0.05 (Figure 5B). To examine whether these proliferation decrease is cell–cell contact dependent, we repeated the proliferation assay with Th17 supernatants instead of Th17 cells and found that the proliferation is still decreased compared with the proliferation of tumour cells alone, but not as potent as with Th17 cells (Figure 5D). The proliferation inhibition cannot be completely abolished with an IL-17 blocking antibody (data not shown).

### Tumour cells express the receptor for IL-17, -21 and -22

Next, we determined by flow cytometry if HNSCC can generally trigger a response to the cytokines secreted by Th17 cells (IL-17, -21 and -22) by expression of the corresponding cytokine receptors of the Th17 cell cytokines (IL-17R, -21R, -22R). We were not able to analyse the IL-26R because of the lack of an appropriate antibody. All tumour cells we have investigated bear the receptors for the Th17 cell cytokines. The IL-17R was found in 93.8±10.2% of tumour cells, IL-21R in 36.7±7.6% and IL-22R in 27.7±9.5% (Figure 5C).

### Th17 cells compromise the angiogenesis of HNSCC

**Figure 6:**
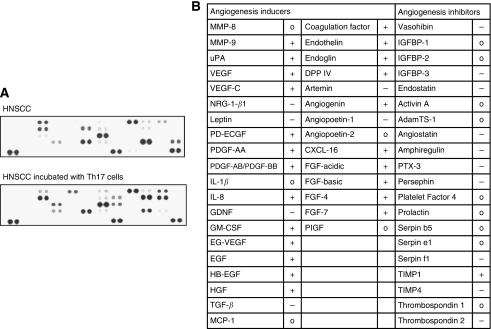
The angiogenesis of HNSCC is impaired in the presence of Th17 cells. Table of the angiogenesis-related proteins spotted on the angiogenesis array. For the angiognesis profiler array, HNSCC cells were incubated over 48 h with or without Th17 cells over 48 h. The protein of the HNSCC cells were then isolated and incubated with the antibody spotted membrane of the angiogenesis array. The first and second columns are the angiogenesis inducers, the third column is the angiogenesis inhibitors (+, upregulation of expression after incubation with Th17 cells; o, no change; −, downregulation of expression).

We analysed whether Th17 cells influence the angiogenesis of HNSCCs. Therefore, we performed a proteome profiler human angiogenesis array to measure the expression of angiogenesis-related molecules in HNSCC in the presence of Th17 cells compared with HNSCC alone. We incubated the HNSCC cell line PCI-13 for 24 h with Th17 cells (concentration 1 : 1) in a transwell chamber and isolated the protein of the tumour cells. We observed an upregulation of most angiogenesis-promoting factors and a downregulation of most angiogenesis-inhibiting factors for the HNSCC cells incubated with Th17 cells compared with HNSCC cells alone (Figure 6).

## Discussion

Less is known about the function of Th17 cells in cancer (Bronte, 2008; Zhang *et al*, 2008a), especially in head and neck cancer. It was shown that Th17 cells are involved in tissue inflammation through the induction of the release of the cytokines IL-8, -6, COX-2, MMP-1, -3, CXCL1 NOS-2 by surrounding cells such as fibroblasts, macrophages, endothelial and epithelial cells (Brennan *et al*, 2001; Agarwal *et al*, 2008; Gu *et al*, 2008), which are involved in angiogenesis, tumour invasion and metastasis (Brennan *et al*, 2001; Nabeshima *et al*, 2002; Li *et al*, 2003; Wei *et al*, 2003). On this account, it is reasonable to propose an impact of Th17 cells on cancer progression and pathogenesis. In ovarian cancer, it was shown that Th17 cells are increased in tumour tissue, but not in peripheral blood (Miyahara *et al*, 2008; Zhang *et al*, 2008a); in gastric cancer, there was both an elevation of Th17 frequency in peripheral blood and TDLNs. But nothing functional is known about Th17 cells and their influence on cancer development in human beings. The aim of our study was to analyse the function of Th17 cells in cancer pathogenesis of HNSCC. First, we studied the frequencies of Th17 cells in peripheral blood, tumour and TDLN tissue of HNSCC patients. We could identify a higher percentage of Th17 cells in peripheral blood of head and neck patients compared with healthy controls. We could not identify any relationship between TNM staging and Th17 cell frequency, so our conclusion is that Th17 cells were consistently elevated in HNSCC patients independent of tumour stage. In addition, all primary tumours except larynx carcinomas and particularly TDLNs are infiltrated by a high number of Th17 cells, whereas TDLNs show a significant higher frequency of Th17 cells than primary tumours. This matter of fact may represent an additional link between the interrelationship of inflammation and cancer, but the exact mechanism of how these high amount of proinflammatory Th17 cells and the therewith connected secreted cytokines contribute to the inflammatory processes in cancer remain to be elucidated. In this article, we report that Th17 cells can be induced through HNSCC supernatants. After incubation of isolated naive CD4+ T cells in head and neck cancer milieu *in vitro*, we could detect a significant elevated number of Th17 cells. We propose that HNSCC microenvironment is, therefore, able to induce Th17 lineage commitment. The HNSCC is a Th17-promoting milieu because of the release of IL-23 and -6 by the tumour cells itself and by TILs and IL-1*β* by tumour-infiltrating immune cells. The IL-1*β* and -6 are required to induce Th17 induction and IL-23 is necessary for the expansion of Th17 cells. We suggest that PGE(2) is a major inducer for Th17 cells in HNSCC microenvironment. We had not examined the amount of PGE(2) in HNSCC tissue, but it is well known that the prostaglandin biosynthesis is impaired in HNSCC, and PGE(2) is highly overexpressed (Camacho *et al*, 2008). Owing to the fact that it was shown that PGE(2) leads to Th17 expansion (Chizzolini *et al*, 2008) and that PGE(2) leading to a rapid increase of ROR*γτ* and the selective enrichment of IL-17-producing cells by modulating the proliferation of memory T cells (Napolitani *et al*, 2009), we could show that PGE(2) levels in HNSCC lead to a strong enhancement of Th17 cell expansion directly at the tumour site and in TDLNs. Next, we found that Th17/Th1 cells were seriously downregulated both in peripheral blood as well as in tumour tissue and TDLNs. It is known that Th1 cytokines are diminished in HNSCC (Bose *et al*, 2008). The IFN*γ* can also be downregulated by TCR triggering in the presence of PGE(2) in memory T cells (Napolitani *et al*, 2009), whereas IL-17 is upregulated, so this maybe the responsible mechanism for the Th17 and Th1/Th17 cell modulation in HNSCC. But the issue remains what happens with this Th17/Th1 cells under tumour influence, if they simply attenuate IFN*γ* secretion or change into another T cell population. Our hypothesis is that Th17 cells were functionally modulated by tumour milieu and were converted into Th1 cells. We could reveal in this article that tumour cells bear all cytokine receptors IL-17R, -21R, -22R necessary to respond on the proinflammaory cytokines secreted by Th17 cells (except IL-26R, which we have not measured), so we propose that tumour cells can be massively influenced by surrounding tumour-infiltrating Th17 cells. Next, we addressed the issue whether Th17 have any functional implications on HNSCC development and HNSCC milieu. First, we analysed whether Th17 cells are able to influence tumour proliferation or even lead to apoptosis of tumour cells. So far, only the influences of the single cytokines from Th17 cells on cancer development were investigated in human beings, but nothing is known about the combinations of neither the Th17 cytokines nor the Th17 cells. There are different reports about the influence of IL-17 on tumour progression, some depict a positive influence of Th17 on tumour proliferation (Benchetrit *et al*, 2002; Numasaki *et al*, 2005) and angiogenesis, whereas others describe a inhibitory influence on tumour growth (Nam *et al*, 2008; Kryczek *et al*, 2009). Muranski *et al* (2008) report that Th17 cells in a mouse model are able to eradicate melanomas. Ciree *et al* (2004) show that IL-17 is upregulated in the T cell lymphomas mycosis fungoides and Sezary syndrome and may act as a tumour growth-promoting or -inhibiting factor. In addition, they show an association between IL-17 expression and polymorphonuclear neutrophil infiltration. This association was affirmed by Garcia-Hernandez Mde *et al* (2010); they describe that neutrophils were attracted to the tumour milieu by an IL-17-dependent mechanism and show that depletion of neutrophils resulted in a diminished capacity to control tumour growth. In addition, Honorati *et al* (2003) report a increased susceptibility of osteosarcoma cells to NK cell lysis under the influence of IL-17. The effects of IL-21 on tumour development are discussed controversially. It was shown that IL-21 not only promotes tumour growth (Menoret *et al*, 2008), but also acts anti-tumourally and inhibited tumour angiogenesis (Castermans *et al*, 2008), enhances antibody-mediated tumour rejection (Smyth *et al*, 2008) and activates NK cells and CD8+ T cells. Opposing views are reported about the regulation of Tregs by IL-21. Lamprecht *et al* (2008) propose an increase of the number of Tregs within tumour microenvironment by inducing CCL20 and therewith the migration of Tregs towards tumour, whereas Kim-Schulze *et al* (2009) revealed a reduction of Tregs through IL-21. Less is known about the function of IL-22 in tumour pathogenesis. Zhang *et al* (2008b) report that autocrine IL-22 has an antiapototic activity in human lung cancer, whereas Weber *et al* (2006) describe an inhibitory effect of IL-22 on signalling pathways promoting cell proliferation. There is no existing data about IL-26 in cancer. In this study, we assessed reduced tumour growth of a head and neck cancer cell line in the presence of different numbers of Th17 cells, whereas there is an induction of pro-angiogenetic factors in tumour cells in the presence of Th17 cells. The decrease of tumour cell proliferation is cell contact dependent because Th17 cell supernatants cannot inhibit the proliferation of HNSCC cells as Th17 cells can inhibit it. But probably there exist a concentration dependency, because we could detect such a dependency in proliferation assay using the cytokine IL-17 (data not shown). We observed a reduced tumour growth with low concentrations of IL-17 and an increase in proliferation with concentrations above 1 pg *μ*l^–1^. Maybe a certain threshold level is needed for tumour cells to use IL-17 for their benefit. In this study, we report that Th17 cells are able to migrate towards HNSCC milieu. We showed that this migration is CCR6/CCL20 driven. It was shown that Th17 cells express CCR6, which enables them to migrate inflamed tissue in response to CCL20. In addition, they express the CCR6 ligand CCL20, though CCL20 from Th17 cells in turn promotes the migration of other Th17 cells (Yamazaki *et al*, 2008). It was shown that different tumour entities overexpress the chemokine CCL20 (Rubie *et al*, 2006a, 2006b) and that the serum levels of patients with nasopharyngeal carcinomas are elevated. In addition, it is established that metastases express more CCL20 than primary tumours (Rubie *et al*, 2006b), so this could be the explanation for the fact that we could measure a higher amount of Th17 cells in lymph node metastasis than in primary tumours. Su *et al* (2010) describe that RANTES and MCP-1 secreted in tumour milieu mediate the recruitment of Th17 cells. In addition, they find that tumour cells and tumour-derived fibroblasts produce a cytokine milieu as well as provide cell–cell contact engagement that facilitates the generation of Th17 cells.

Altogether, we showed for the first time that Th17 cells are present to a high amount in HNSCCs. In addition, Th17 cells were induced through factors in tumour milieu and actively migrate towards tumour milieu and have a potent impact on carcinogenesis of head and neck cancers. However, it is similar to an double-edged sword, on the one side Th17 cells seem to be beneficial to the host because of their proliferation reducing activity, on the other side they accelerate metastasis through the induction of angiogenesis and seem, therefore, beneficial to tumour. Therefore, it is important to study the function of Th17 cells in malignant diseases in depth and try to elucidate their mechanism of action and their modulation by tumour microenvironment to find a possibility how Th17 cells have to be modulated that they are beneficial for tumour patients. Our results raise several further issues to investigate – first, whether Th17 cells express different molecules or secrete different cytokines in HNSCC patients compared with healthy persons. Eventually, it has to be investigated in future whether Th17 cells can recognise tumour cells and if they are at all able to impair tumour growth or metastasis *in vivo* and if they do so, one has to elucidate the exact mechanism of action. Another open question is whether HNSCC is able to modulate Th17 function for immune escape.
